# Predictors of necessity for endoscopic balloon dilatation in patients with Crohn’s disease-related small bowel stenosis

**DOI:** 10.1080/07853890.2021.1998597

**Published:** 2021-11-09

**Authors:** Yukie Hayashi, Kaoru Takabayashi, Naoki Hosoe, Hiroki Kiyohara, Satoshi Kinoshita, Kosaku Nanki, Kayoko Fukuhara, Yohei Mikami, Tomohisa Sujino, Makoto Mutaguchi, Takaaki Kawaguchi, Motohiko Kato, Haruhiko Ogata, Takanori Kanai

**Affiliations:** a Division of Gastroenterology and Hepatology Department of Internal Medicine, School of Medicine, Keio University, Tokyo, Japan; b Center for Diagnostic and Therapeutic Endoscopy, School of Medicine, Keio University, Tokyo, Japan; c Center for Preventive Medicine, Keio University Hospital, Tokyo, Japan

**Keywords:** Crohn’s disease, small bowel stenosis, endoscopic balloon dilatation

## Abstract

**Background and Aim:**

In patients with Crohn’s disease (CD) and small bowel stenosis, endoscopic balloon dilation (EBD) is considered to be useful in improving stenotic symptoms and avoiding surgery. However, it carries risks such as bleeding and perforation. The aim of this study was to identify the indications for endoscopic intervention in patients with CD and small bowel stenosis.

**Methods:**

From November 2007 to March 2020, 143 CD patients with small bowel stenosis were enrolled in this study. We identified the factors associated with not requiring endoscopic intervention during long-term follow-up of these patients.

**Results:**

Forty of the 143 patients had abdominal symptoms of stenosis and had undergone EBD, whereas the remaining 103 were asymptomatic and had not undergone endoscopic intervention. During long-term follow-up, 95 of those 103 patients never required endoscopic or surgical intervention. Multivariate logistic regression analysis revealed that not consuming an elemental diet (OR 3.18, 95% CI 1.48–6.82; *p* < .01) and ileocecal valve (ICV) stenosis (OR 0.30, 95% CI 0.11–0.83; *p* = .02) were independently associated with not requiring EBD. The cumulative emergency hospitalisation-free rate also tended to be higher in patients not consuming an elemental diet or with ICV stenosis.

**Conclusions:**

Two factors, namely not consuming an elemental diet and ICV stenosis, predict a long-term intervention-free prognosis in CD patients with small bowel stenosis.

**Key messages**
When an endoscopically impassable small bowel stenosis is found in a CD patient, long-term follow-up without endoscopic intervention may be possible if the patient is asymptomatic, is not using an elemental diet, and the stenosis is ICV.

## Introduction

Crohn’s disease (CD), an inflammatory bowel disease, can cause granulomatous inflammatory lesions with ulceration and fibrosis through the gastrointestinal tract. Small bowel stenosis is one of the serious complications of CD. In fact, approximately half of CD patients develop stenosis and fistulas within 5 years and 50% of patients require surgery within 10 years [1].

Numerous studies have reported that endoscopic balloon dilatation (EBD) is a safe and effective means of improving stenotic symptoms and avoiding surgery in patients with CD [2–9]. Although EBD is a less-invasive procedure than surgery, it is not advisable to perform EBD in all patients with small bowel stenosis because of risks such as bleeding [9], perforation [4], abscess, and fistula [10,11]. However, the indications for EBD in CD patients with small bowel stenosis are yet to be clarified.

As noted above, although many groups have reported on the benefits of EBD in CD patients with small bowel stenosis, only a few have examined risk factors for recurrence of stenosis [7,12]. However, all patients in one study had undergone EBD and only stenosis at the anastomosis was studied [7]. Another study only included symptomatic patients who had not previously undergone surgery [12]. Patients who have previously undergone surgery and/or have asymptomatic small bowel stenosis are often encountered in clinical practice. Indeed, the need for endoscopic treatment of incidentally found stenoses in asymptomatic patients is controversial [10]. To date, there is no clear evidence for treating small bowel stenosis in symptomatic or asymptomatic CD patients with stenosis.

Small bowel stenosis is a clinically important problem for patients with CD, but small bowel barium follow through and MR enterography to evaluate the entire small bowel are not available in all centres and are difficult to perform annually. In fact, in many clinical situations, the entire small bowel is not always evaluated routinely, and the small bowel is evaluated by colonoscopy using Simple endoscopic score for Crohnʼs disease (SES-CD) [13] or by balloon-assisted enteroscopy (BAE) as far as it can be observed. Furthermore, if the patient has no symptoms of stenosis, the patient may be followed up without EBD even if there is small bowel stenosis. Therefore, this study was designed to investigate the predictors of CD patients requiring endoscopic intervention for small bowel stenosis by following the course of patients with even one endoscopically impassable stenosis in the area observable by BAE.

## Patients and methods

### Patients and study design

**Figure 1.**
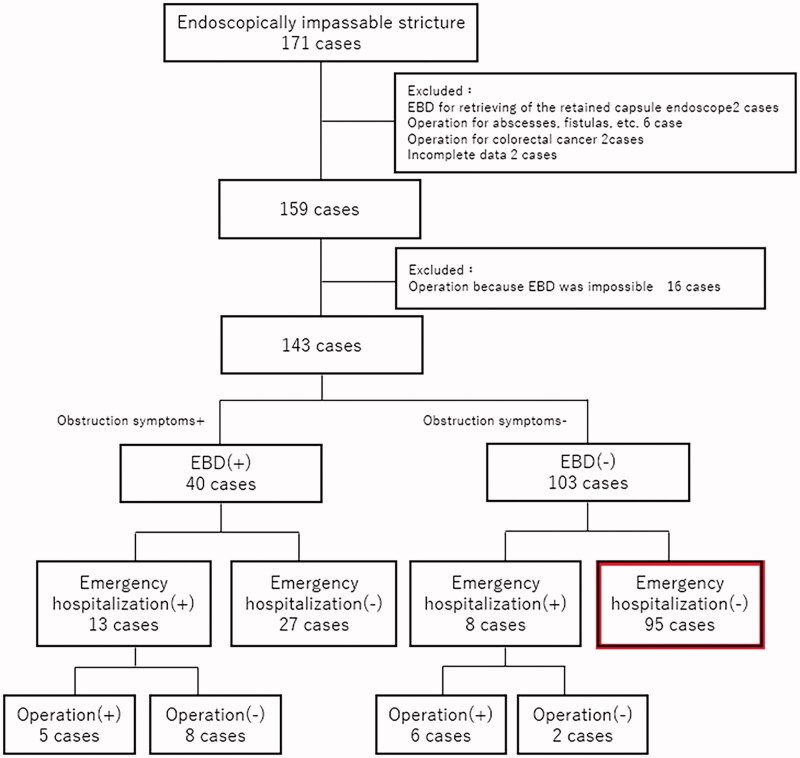
Flowchart of study. Emergency hospitalisation: emergency admission for gastrointestinal stenosis requiring therapeutic interventions such as decompression, balloon dilation, or surgery. Red squares: patients who had not undergone EBD and did not relapse. EBD: Endoscopic balloon dilation.

The cohort of this retrospective study comprised 171 patients with ileal or ileocolonic types of CD and small bowel stenosis through which an endoscope (SIF-Q260; Olympus, Tokyo) could not be passed. In all patients, barium contrast was performed from the endoscopically impassable small bowel stricture to the oral small bowel, and the stricture length was assessed. These patients were identified by searching records of 424 CD patients who had undergone trans-anal enteroscopy at Keio University Hospital between November 2007 and March 2020. To facilitate assessment of the prognosis of small bowel stenosis, we excluded 12 patients who were scheduled for surgery for stenosis, or had undergone EBD to retrieve a retained capsule endoscope, or had undergone surgery for conditions other than stenosis, such as abscesses, fistulas, or colorectal cancer, or for whom insufficient medical data had been recorded. We also excluded 16 patients who underwent surgery for inadequate EBD of small bowel stricture. The remaining 143 CD patients with small bowel stenosis were enrolled in the analysis (Figure 1) and any subsequent emergency hospitalisations identified by searching their medical records.

The following characteristics of eligible patients were obtained from medical records: as age, sex, duration of CD, disease type, surgical history, blood test data (The blood test data is the value when the patient was first diagnosed with stenosis by surveillance endoscopy), date of diagnosis of stenosis, site of stenosis, Harvey–Bradshaw index (HBI) at the time of diagnosis of stenosis, balloon dilatation performed, medications used, history of emergency hospitalisation for gastrointestinal stenosis requiring therapeutic interventions such as decompression or balloon dilatation/surgery, and surgery for gastrointestinal stenosis after diagnosis of that stenosis.

In our hospital, barium contrast was performed from the deepest part of the small bowel that could be reached by the enteroscopy to evaluate the small bowel on the mouth side as much as possible. Not all patients underwent barium or CT scans of the small intestine, but all lesions that could be observed by endoscopy were evaluated as much as possible. When EBD was performed for stenosis, the basic goal was to dilate the stenosis to 12–15 mm so that it could be passed through the endoscope. However, this was not the case when it was judged difficult to dilate to the goal due to mucosal laceration or bleeding.

### Endpoints and definitions

The primary endpoint was identification of predictors of the need for endoscopic intervention in CD patients with small bowel stenosis. The secondary endpoint was determining whether cumulative emergency hospitalisation-free rates were significantly associated with these predictors. Emergency hospitalisation was defined as emergency admission for small bowel stenosis requiring a therapeutic intervention such as decompression, balloon dilatation, or surgery after diagnosis of stenosis. We defined constriction symptoms as abdominal pain, abdominal bloating, and nausea, and asymptomatic as the absence of these symptoms. Our policy is to perform EBD on patients with symptomatic stenosis during a scheduled admission, whereas asymptomatic patients are followed up without EBD. Therefore, we did not classify scheduled admissions to perform EBD as emergency hospitalisation. Furthermore, to facilitate identification of factors associated with not needing endoscopic intervention during long-term follow-up, we allocated the 143 study patients to therapeutic intervention or non-therapeutic intervention groups. The therapeutic intervention group was defined as all patients who had undergone EBD, including some who required emergency hospitalisation for stenotic symptoms, and those who had not undergone EBD but required emergency hospitalisation during follow-up.

### Ethical considerations

This retrospective study was approved by the ethics committee of Keio University Hospital (Approval No. 20160431).

### Statistical analysis

JMP software version 14.0 (SAS Institute, Cary, NC, USA) was used for all statistical analyses. Continuous variables are expressed as mean (minimum–maximum). As for continuous variables, Student's *t*-test was used to compare parametric variables between the two groups for and the Mann–Whitney *U* test for non-parametric variables. The *χ*^2^ test was used to assess nominal variables. To analyse the characteristics of patients requiring intervention for stenosis, multivariate analysis was also performed by logistic regression analysis, using four factors identified by stepwise regression. Kaplan–Meier analysis and the log-rank test were used to analyse cumulative emergency hospitalisation-free rate. *P*-values < .05 were defined as denoting significant differences.

## Results

### Patients characteristics and clinical outcomes

Background characteristics of the 143 patients are shown in Table 1. The male to female ratio was 112–31 and the mean disease duration of CD was 13.7 years. The mean age at diagnosis of stenosis was 40.1 years and 91 patients had previously undergone surgery. The median HBI was 4, mean albumin concentration 3.75 g/dL, and mean C-reactive protein (CRP) concentration 0.92 mg/dL. Anti TNF-α antibody had been administered to 55.9% of the patients and 42.7% of them were on elemental diets. The stenoses were in the small bowel in 49 patients, the ileocecal valve (ICV) in 44, and anastomoses in 50. The mean duration of follow-up was 1641.0 days. Forty of the 143 patients had abdominal symptoms and underwent EBD; the remaining 103 patients were asymptomatic and were simply followed up. Forty patients underwent scheduled EBD after being diagnosed with small bowel stenosis, 13 of whom required emergency hospitalisation. In contrast, eight of the 103 asymptomatic patients who had not undergone EBD subsequently required emergency hospitalisation.

**Table 1.** Patient characteristics.

Characteristics	
Number of patients	143
Gender (male/female)	112/31
Age at diagnosis of stricture (years), mean	40.1 (18–81)
Disease duration (years), mean	13.7 (0–43)
Disease location (ileal/ileocolonic)	28/115
History of surgery (yes/no)	91/52
Harvey-Bradshaw index, median	4 (0–22)
Medications	
5-amynosalicylic acid	108 (75.5%)
Azathioprine	24 (16.8%)
6-mercaptoprine	36 (25.2%)
Anti TNF-α antibody	80 (55.9%)
Elemental diet	61 (42.7%)
(<900ml/day / ≧900ml/day)	34 (23.8%)/27 (18.9%)
Steroids	7 (4.9%)
Blood examinations	
Albumin (g/dl)	3.75 (1.7–5)
C-reactive protein (mg/dl)	0.92 (0.01–16.4)
Location of strictures (small intestine/ileocecal valve/anastomosis)	49/44/50
Length of strictures (<1cm/≧1cm)	102/41
Ulcer on stricture (yes/no)	87/56
Endoscopic balloon dilation (no/one time/multiple times)	103/28/12
Addition or change of anti TNF-α antibody after diagnosis of strictures	24 (16.8%)
Addition or change of immunomodulator after diagnosis of strictures	10 (7.0%)
Observation period (days), mean	1641.0 (8–4549)

The characteristics of the 40 patients who underwent EBD and those who did not are shown in Supplementary table 1. There was a significant difference in disease location and location of strictures. Patients who received EBD were significantly more likely to use the Elemental diet.

### Risk factors for emergency hospitalisation in patients who underwent EBD

In the subgroup of 40 patients who had undergone EBD, we assessed differences in the characteristics of the 13 patients who required emergency hospitalisation and the 27 who did not (Table 2). We found no significant differences between the two groups for most investigated factors, the only exception being significantly higher CRP concentrations in the group requiring emergency hospitalization (*p* = .04). There was no significant difference in HBI between the emergency hospitalisation group and the non-emergency hospitalisation group, and there was no significant difference even when comparing each item of HBI.

**Table 2.** Characteristics of patients who had undergone EBD according to emergency hospitalisation status.

Characteristics	Emergency hospitalisation	Non-emergency hospitalisation	*p* value
Number of patients	13	27	–
Gender (male/female)	10/3	23/4	.53^†^
Age at diagnosis of stricture (years), mean	39.7 (21–63)	43.3 (24–81)	.60^‡^
Disease duration (years), mean	16.5 (2–37)	14.9 (1–43)	.65^§^
Disease location (ileal/ileocolonic)	2/11	11/16	.10^†^
History of surgery (yes/no)	11/2	19/8	.32^†^
Harvey-Bradshaw index, median	4.23 (1–7)	4.19 (0–11)	.21^‡^
Medications			
5-amynosalicylic acid	11 (84.6%)	20 (74.1%)	.44^†^
Azathioprine	2 (15.4%)	7 (25.9%)	.44^†^
6-mercaptoprine	3 (23.1%)	8 (29.6%)	.66^†^
Anti TNF-α antibody	6 (46.2%)	16 (59.3%)	.44^†^
Elemental diet	9 (69.2%)	18 (66.7%)	.87^†^
(≧900ml/day)	3 (23.1%)	9 (33.3%)	.50^†^
Steroids	2 (15.4%)	2 (7.4%)	.44^†^
Blood examinations			
Albumin (g/dl)	3.73 (2.4–4.6)	3.84 (2.0–4.9)	. 85^‡^
C-reactive protein (mg/dl)	1.22 (0.01–5.00)	0.44 (0.01–3.43)	.04^§^
Location of strictures (small intestine/ileocecal valve/anastomosis)	5/0/8	12/3/12	.23^†^
Length of strictures (<1cm/≧1cm)	7/6	22/5	.07^†^
Ulcer on stricture (yes/no)	10/3	15/12	.18^†^
Balloon diameter (12/13.5/15/18 mm)	5/5/2/1	8/12/7/0	.39^†^
Addition or change of anti TNF-α antibody after diagnosis of strictures (yes/no)	1/12	4/23	.51^†^
Addition or change of immunomodulator after diagnosis of strictures (yes/no)	2/11	1/26	.21^†^

^†^*χ*^2^-test.

^‡^Mann–Whitney *U* test.

^§^Student’s *t-*test.

### Characteristics of patients who did not require intervention for stenosis

Emergency hospitalisation was not required during follow-up in 95 of the patients who had not undergone EBD (Figure 1). We compared the clinical characteristic of these 95 patients, who did not have emergency hospitalisations without interventions such as EBD, surgery or decompression (non-therapeutic intervention group), with those of the other 48 patients (therapeutic intervention group) (Table 3). We found that patients who did not require therapeutic interventions had significantly less history of surgery (*p* = .04), lower HBI scores (*p* = .01), and were less likely to be on elemental diets (*p* < .01) than those who did require therapeutic interventions. Additionally, patients who did not require therapeutic intervention were significantly more likely to have stenoses in ICVs than in the small bowel or an anastomosis (*p* < .01). We also compared patients with ICV stenosis between those who underwent EBD and those who did not, but found no significant differences in other clinical characteristics such as stenosis length or presence of ulcers.

**Table 3.** Patient characteristics according to requirement for therapeutic intervention.

Characteristics	Therapeutic intervention^§^	Non-therapeutic intervention	*p* value
Number of patients	48	95	–
Gender (male/female)	38/10	74/21	.86^†^
Age at diagnosis of stricture (years), mean	41.3 (21–81)	39.5 (18–77)	.77^‡^
Disease duration (years), mean	15.1 (1–43)	13.0 (0–41)	.19^‡^
Disease location (ileal/ileocolonic)	13/35	15/80	.11^†^
History of surgery (yes/no)	36/12	55/40	.04^†^
Harvey-Bradshaw index, median	4.77 (0–13)	3.99 (0–22)	.01^‡^
Medications			
5-amynosalicylic acid	38 (79.2%)	70 (73.7%)	.47^†^
Azathioprine	9 (18.8%)	15 (15.8%)	.66^†^
6-mercaptoprine	15 (31.3%)	21 (22.1%)	.24^†^
Anti TNF-α antibody	29 (60.4%)	51 (53.7%)	.44^†^
Elemental diet	31 (64.6%)	30 (31.6%)	<.01^†^
(≧900ml/day)	12 (25.0%)	15 (15.8%)	.19^†^
Steroids	5 (10.4%)	2 (2.1%)	.03^†^
Blood examinations			
Albumin (g/dl)	3.71 (1.7–4.9)	3.77 (1.7–5.0)	.95^‡^
C-reactive protein (mg/dl)	0.99 (0.01–5.76)	0.88 (0.02–16.4)	.97^‡^
Location of strictures (small intestine/ileocecal valve/anastomosis)	19/6/23	30/38/27	<.01^†^
Length of strictures (<1cm/≧1cm)	30/18	72/23	.10^†^
Ulcer on stricture (yes/no)	31/17	56/39	.51^†^
Addition or change of anti TNF-α antibody after diagnosis of strictures (yes/no)	9/39	15/80	.66^†^
Addition or change of immunomodulator after diagnosis of strictures (yes/no)	4/44	6/89	.66^†^

^†^*χ*^2-^test.

^‡^Mann–Whitmey *U* test.

^§^Therapeutic intervention group: all patients who had undergone EBD for stenotic symptoms or emergency hospitalisation for decompression, balloon dilatation, or other surgeries for symptomatic stenosis. The latter group includes eight patients who had not undergone EBD.

Multivariate analysis was performed using logistic regression to identify factors associated with the need for interventions for stenoses (Table 4). This showed that stenoses of ileocecal valves (OR 0.30, 95% CI 0.11–0.83; *p* = .02) and not consuming an elemental diet (OR 3.18, 95% CI 1.48–6.82; *p* < .01) were independently associated with not requiring interventions during follow-up.

**Table 4.** Factors associated with need for therapeutic intervention for stenosis.

Characteristics	Multivariate HR(95%CI), *p* value
History of surgery	1.59 (0.47–2.77), .76
Harvey-Bradshaw index (>4)	1.58 (0.72–3.48), .25
Medications Elemental diet	3.18 (1.48–6.82), <.01
Location of strictures (ileocecal valve)	0.30 (0.11–0.83), .02

Logistic regression analysis.

### Characteristics associated with cumulative emergency hospitalisation-free rate

**Figure 2.**
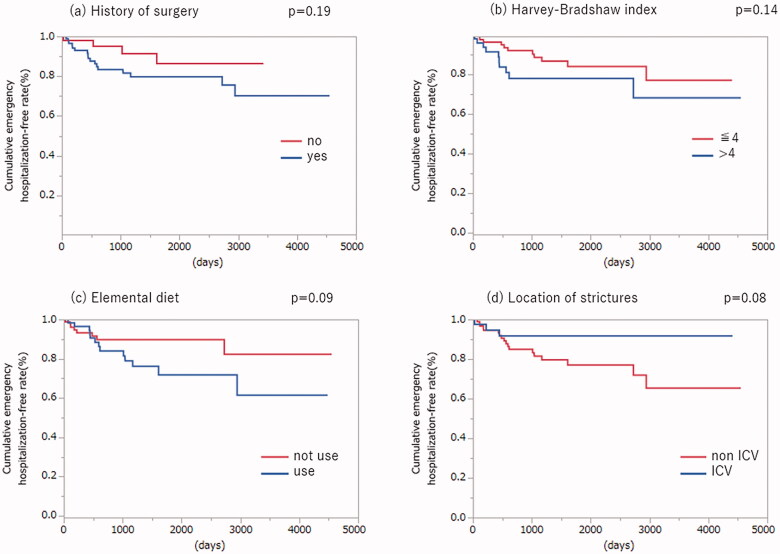
Cumulative emergency hospitalisation-free rate for all patients. Kaplan–Meier curves depicting cumulative emergency hospitalisation-free rate stratified by history of surgery (a), Harvey–Bradshaw index (b), consumption of elemental diet (c), and location of stricture (d). *p* values on each curve were calculated using the log-rank test.

We performed Kaplan–Meier analysis of cumulative non-emergency hospitalisation for the four factors extracted by stepwise regression shown in Table 4: surgical histories, HBI, consumption of elemental diet, and site of stenosis (Figure 2). We noted a trend towards fewer non-emergency hospitalisations in patients not consuming elemental diets and with ICV stenoses; however, these differences were not statistically significant. No trends were identified regarding surgical histories and HBI.

## Discussion

This study was designed to identify predictors of the need for EBD in CD patients with small bowel stenosis and factors associated with emergency hospitalisation for bowel obstruction.

First, we compared background factors among patients who had undergone EBD. The only factor that differed significantly between patients who did and did not require emergency hospitalisation was CRP concentration (Table 2). This finding suggests that residual intestinal inflammation leads to future hospitalisation even after the patient has undergone EBD. Therefore, disease activity should be reduced as much as possible before performing EBD in symptomatic patients.

As shown in Figure 1, 103 of 143 CD patients with small bowel stenosis were asymptomatic and did not undergo EBD. Additionally, 95 of those who had not undergone EBD did not require emergency hospitalisation for intestinal obstruction during follow-up. Although a prospective study in the context of the patient population is of course necessary, these results may suggest that EBD is not always necessary for asymptomatic patients with endoscopic stenosis. Previous studies have reported benefits of EBD in patients with CD-related stenosis regardless of presence of symptoms [4–9,14–16] or in symptomatic patients [12]; however, to our knowledge, no studies have separately assessed outcomes of EBD in patients with asymptomatic small bowel stenosis. Given that EBD carries risks such as bleeding and perforation, EBD may not be indicated in asymptomatic patients.

As many of the patients who had not undergone EBD had not required hospitalisation (95 patients), we compared the characteristics of patients who had not undergone EBD and did not require emergency hospitalisation with those of the remaining patients (Table 3). We found that patients with histories of surgery, higher HBI scores, on elemental diets, use of steroids, and with stenoses at sites other than ICVs were at significantly higher risk of emergency hospitalisation. Additionally, consumption of elemental diets and non-ICV stenoses remained significant factors after multivariate analysis (Table 4).

Elemental diets are often prescribed when patients report stenotic symptoms or when intestinal rest is required. In our hospital, patients who had stenosis symptoms or activity in the past and were able to achieve remission by combining elemental diet with other treatments have continued to use elemental diet with the aim of maintaining remission. Therefore, consumption of an elemental diet may potentially indicate the presence of lesions that are not currently reflected in CRP concentrations but may be responsible for stenosis symptoms. In fact, the administration of elemental diet may involve somewhat subjective judgement by the physician, but since elemental diet are not prescribed for patients with no stenosis symptoms at all, it is one indicator of the presence of stenosis symptoms. Additionally, histories of surgery, high HBI scores, and use of steroids likely indicate very active disease. Thus, EBD or intensification of treatment is probably indicated in patients on elemental diets with stenotic symptoms, because the use of elemental diet alone is limited in maintaining remission. In support of this contention, Ding et al. have also reported that patients with very active disease are more likely to require surgery [7]. Those authors stated that escalation of medication reduces the risk of restenosis. Bamba et al. have also reported significantly higher surgery-free survival rates in patients who are treated with immunomodulators or anti-tumor necrosis factor (TNF)-α antibodies after the onset of symptoms [12]. Furthermore, in a prospective study by Bouhnik et al., adalimumab showed short-term efficacy for small bowel stricture, in particular short and not severe stricture without fistula [17]. However, in the present study, we did not find that addition of immunomodulators or anti-TNF-α antibodies had a significant effect on rates of emergency hospitalisation. Drug treatment for small bowel stenoses in CD patients is controversial. Some researchers have reported that infliximab administration is effective [18–20], whereas others have found it is not [21,22]. As for the reason for the different results, the objects of this study originally had a high introduction rate of anti-TNF, so it is possible that the effect of additional treatment using anti-TNF was not shown. Prospective studies are needed to clarify this issue.

The low incidence of emergency hospitalisation in patients with ICV stenosis may be attributable to this site’s flexibility. Several previous studies have shown that endoscopic submucosal dissection (ESD) or endoscopic mucosal resection (EMR) for neoplasia in the ICV region rarely causes stenotic symptoms [23–25]. It has been suggested that ICVs are less prone to stenosis than other regions because ICVs are regularly stretched by the passage of stools and are comprised of more flexible tissue than other parts of the gastrointestinal tract. Thus, although CD is not the same condition as after mucosal resection because the disease causes inflammation in all layers of the intestine, a relatively soft and asymptomatic stricture may form in the ICV during the healing process of inflammation. This suggests that EBD may not always be necessary for patients with asymptomatic ICV stenosis, although prospective studies are needed. In fact, Takabayashi et al. reported that the activity of the terminal ileum was not associated with prognosis [26]. Therefore, although it is useful to observe the deep small intestine to assess future prognosis, there is no need to dilate the ICV just to improve stenosis symptoms.

In the current study, we examined associations between factors detected by our analysis and long-term prognosis of CD patients with small bowel stenosis. Although these associations are not statistically significant, our findings suggest that patients in two categories, namely not consuming an elemental diet and ICV stenosis, are unlikely to require emergency hospitalisation and can therefore be followed up without undergoing EBD. In fact, six of the eight patients who did not undergo EBD but later required emergency hospitalisation were consuming an elemental diet use or had a non-ICV stenosis. We therefore concluded that consuming an elemental diet and non-ICV stenosis are likely risk factors for requiring emergency hospitalisation for stenosis.

This study has several limitations. First, it was a retrospective study. Because the information was not collected prospectively, we could not explore possible confounding factors that were not documented in the medical records, such as intensity of symptoms, pre-existing disease, and lifestyle habits. One study has reported that smoking may be associated with the need for EBD [12]. However, we could not evaluate the effects of a smoking habit on requirement for EBD in this study. Secondly, we included only patients who had undergone transanal enteroscopy and barium contrast with endoscopy from the deepest part of the bowel. Only some of the patients had undergone small bowel barium follow through for entire intestine or computed tomography (CT). This suggests that endoscopically detectable stenoses are not always the most significant lesions. Thirdly, the study cohort was relatively small. Because this was a single-center study and only included CD patients with small bowel stenoses, it was difficult to recruit a sufficient number of eligible patients. A multicenter, prospective study is necessary to obtain clear evidence.

In conclusion, in this study we identified the characteristics of CD patients who may not require intervention for stenosis. Two factors, not consuming an elemental diet and ICV stenosis, predict a long-term intervention-free prognosis in CD patients with small bowel stenosis.

## Supplementary Material

Supplemental Material

## Data Availability

The data that support the findings of this study are available from the corresponding author, K.T., upon reasonable request.
