# Supplementary material for: Predictors of necessity for endoscopic balloon dilatation in patients with Crohn’s disease-related small bowel stenosis
Source: Ann Med. 2021 Nov 9;53(1):2025–33. doi: 10.1080/07853890.2021.1998597 (PMC8583773; doi:10.1080/07853890.2021.1998597)
Supplement: Supplemental Material [file IANN_A_1998597_SM6209.docx]

**Supplementary table 1. Characteristics of patient with/without EBD**

| Characteristics | EBD(+) | EBD(-) | p value |
| --- | --- | --- | --- |
| Number of patients | 40 | 103 | - |
| Gender (male/female) | 33/7 | 79/24 | 0.45^†^ |
| Age at diagnosis of stricture (years), mean | 42.2(21-81) | 39.3(18-77) | 0.50^‡^ |
| Disease duration (years), mean | 15.4(1-43) | 13.1(0-41) | 0.20^‡^ |
| Disease location (ileal/ileocolonic) | 12/28 | 15/88 | 0.04^†^ |
| History of surgery (yes/no) | 30/10 | 61/42 | 0.07^†^ |
| Harvey-Bradshaw index, median | 4.21(0-11) | 4.26(0-22) | 0.13^‡^ |
| Medications  5-amynosalicylic acid  Azathioprine  6-mercaptoprine  Anti TNF-α antibody  Elemental diet  (≧900ml/day)  Steroids | 31(77.5%)  9(22.5%)  11(27.5%)  22(55.0%)  27(67.5%)  12(30.0%)  4(10.0%) | 77(74.8%)  15(14.6%)  25(24.3%)  58(56.3%)  34(33.0%)  15(14.6%)  3(2.91%) | 0.73^†^  0.25^†^  0.69^†^  0.89^†^  <0.01^†^  0.04^†^  0.08^†^ |
| Blood examinations  Albumin (g/dl)  C-reactive protein (mg/dl) | 3.81(2.0-4.9)  0.70(0.01-5.00) | 3.73(1.7-5.0)  1.01(0.01-16.4) | 0.30^‡^  0.15^‡^ |
| Location of strictures  (small intestine/ileocecal valve/anastomosis) | 17/3/20 | 32/41/30 | <0.01^†^ |
| Length of strictures (<1cm/≧1cm) | 29/11 | 73/30 | 0.85^†^ |
| Ulcer on stricture (yes/no) | 25/15 | 62/41 | 0.80^†^ |
| Addition or change of anti TNF-α antibody after diagnosis of strictures (yes/no) | 5/35 | 19/84 | 0.39^†^ |
| Addition or change of immunomodulator after diagnosis of strictures (yes/no) | 3/37 | 7/96 | 0.88^†^ |

^†^χ^2^ test

^‡^Mann–Whitmey U test
